# Supplementary material for: Are carbon emissions trading and green financial instruments synergistic? -Comprehensive quantitative research based on content analysis
Source: PLoS One. 2024 Mar 7;19(3):e0298601. doi: 10.1371/journal.pone.0298601 (PMC10919601; doi:10.1371/journal.pone.0298601)
Supplement: S1 Data — (ZIP) [file pone.0298601.s001.zip › Data/地级市Z_F工作报告.zip!/║■▒▒/╢⌡╓▌╩╨/└┤╫╘╣1⁄2╓┌║┼í╛┬φ┐╦╩2╛▌═°í┐_2019.docx]

各位代表：

现在，我代表市人民政府向大会报告工作，请予审议，并请各位市政协委员和列席人员提出意见。

　一、2018年工作回顾

刚刚过去的2018年，是全面贯彻党的十九大精神的开局之年，也是改革开放40周年。一年来，我们坚持以习近平新时代中国特色社会主义思想为指导，牢记习近平总书记“四个切实”殷殷嘱托，在省委、省政府和市委的坚强领导下，坚持稳中求进工作总基调，坚持新发展理念，按照高质量发展要求，围绕打造湖北特色产业增长极，统筹推进“稳促调惠防”各项工作，实现了经济社会持续健康发展。

——经济总量实现新跨越。全市实现地区生产总值1005.3亿元，比上年增长8.2%，首次突破千亿元大关。规模以上工业增加值增长7.4%，全社会消费品零售总额增长12.5%，一般公共预算收入增长9.3%，税收占一般公共预算收入比重为70.7%，比上年提高8.7个百分点。主要指标增幅好于全国、全省平均水平。

　——三大攻坚开局良好。金融风险点有效控制，着力整治非法集资、互联网金融；积极防范化解政府债务风险，政府负债率在合理范围内。精准脱贫深入推进，15775人脱贫、6个村出列。污染防治成效明显，可吸入颗粒物（PM10）、细颗粒物（PM2.5）平均浓度分别下降13.1%、17.9%，空气质量优良天数比例达75.2%；地表水上级考核断面及县级以上集中式饮用水源地水质达标率均为100％。

——基础设施不断完善。全市基础设施投资增长7.7%，其中主城区累计完成基础设施建设投资52亿元，增长21%。持续推进全国文明城市创建；凤凰路交通畅通工程、六中地下通道等工程实施，城市交通进一步改善；社区建设“40工程”加快推进，城市功能进一步完备；各区、开发区实施了一批市政基础设施工程，城市建设迈上新台阶。

——民生福祉稳步提升。财政投入民生支出占比75.6％。城镇、农村常住居民人均可支配收入达31751元、17510元，分别增长8%、8.3%。开工棚户区改造12704套，基本建成7799套，完成情况居全省前列。城镇新增就业20565人，城镇登记失业率2.3%。市场物价总水平基本稳定。全面小康加快推进，更多群众分享到发展成果。

事非经过不知难。过去的一年，改革发展稳定任务之重前所未有，矛盾风险挑战之多前所未有，稳增长抓保护考验之大前所未有。尤其是面临全球主要经济体增长放缓、贸易保护主义抬头等不利因素，国内经济运行下行压力加大，宏观经济环境趋紧。在巨大压力和严峻挑战面前，全市上下团结拼搏、攻坚克难、沉着应对，创造了经济社会发展不平凡的业绩。实践再次证明，任何艰难险阻都挡不住全市人民坚毅前行的步伐！

一年来，主要做了以下五个方面的工作：

　（一）立足稳增长，加快产业结构转型升级

工业经济提质增效。抓好工业企业运行协调，组织银企对接活动，加强要素保障服务，为工业企业稳增长创造良好环境。深化供给侧结构性改革，巩固钢铁水泥行业化解产能成果，单位GDP能耗下降5%以上。实施质量强市战略，加强品牌创建，不断提高产品市场竞争力。多措并举扶持小微企业发展，税收过亿元企业8家，新增产值过10亿元企业3家。规模以上工业企业利润增长67.6%，增速居全省前列。鄂钢生产的桥梁钢市场占有量连续三年全国第一。16家企业入选全省支柱产业细分领域隐形冠军企业。成功注册商标785件，商标有效注册总量超过4000件。

新动能培育加快。坚持“育新”与“优旧”相结合，实施百家企业技改工程，工业投资增长14.5%，其中，技改投资增长18.4%，民间投资增长12.3%。支持各类市场主体发展，优化民营经济发展环境，新登记各类市场主体1.48万户，增长10.5%。高新技术产业增加值增长10.5%。实施“新鄂州人”计划，大力推进“121”人才池建设，引进急需人才108名，万人发明专利拥有量4.12件，居全省第三。虹润新材料一期、南都新能源一期、容百锂电三期等项目建成投产，百亿级新能源产业已现雏形。电厂三期并网发电。葛店国家级开发区综合排名由2016年全省垫底提升至第三。华容区获全省县域经济发展进位奖。

现代服务业迅猛发展。把服务业作为城市转型、实现高质量发展的主要抓手，设立1亿元产业发展基金，精准有效支持电子商务、科技服务等服务业企业快速成长。唯品会、苏宁易购等电商企业继续发挥重要支撑作用。高技术服务业较快发展，葛店开发区获批建设国家级检验检测高技术服务业集聚区。服务业增加值增速持续领跑全省，占GDP比重提高2.5个百分点，再创历史新高。金融业态势良好，全市金融机构本外币存款余额和贷款余额分别增长10.7%、14.3%。

（二）聚焦抓项目，厚植高质量发展基础

机场建设前期工作全面加快。举全市之力推进鄂州机场建设。机场已于去年2月9日获国务院、中央军委批复。机场报批工作已完成选址、预可研、可研和机场总规审批手续，初步设计报批进入最后冲刺阶段。鄂州海关成功获批。协调解决了机场净空处理区取土、花马湖水面经营权回购等一系列重大难题，有效保障了走马湖水系综合治理、机场快速通道（S203鄂州段）、配套电力工程、安置小区等系列工程顺利推进。

招商引资工作势头良好。坚持产业方向，坚持招大引强，坚持领导挂帅，强力推进招商引资工作。签约亿元以上项目77个，合同投资额716亿元，其中10亿元以上项目4个，50亿元以上项目1个，100亿元以上项目3个。铠尔慷智能装备制造、富鸿鑫电子、枫树线业产业园、容百锂电正极材料生产基地、融创体育小镇等重大项目签约落户。开工亿元以上项目61个。完成省外资金295亿元。在第八届亚洲物流及航运会议中举办鄂州专场论坛，鄂州空港全球推介跨出重要一步。

基础设施建设积极推进。坚持强功能、补短板、提品质，加大城建投资力度。控违查违“铁拳行动”拆除违法建设24.67万平方米，创历史新高。马鞍山和孙权大道地下综合管廊、老城区雨污分流改造等市政工程顺利实施；梁子湖水系连通及堤防加固、花马湖二站等水生态保护工程稳步推进。市民中心、市图书馆投入使用，鄂州综合客运枢纽站试运营，雨台山水厂改扩建完工，110千伏文星变电站竣工，互联网普及率全省第二。鄂咸高速加快推进，武汉地铁11号线葛店段获国家发改委批复。三江港疏港联运铁路主体工程完工，综合码头一期即将开港，多式联运工程入选国家多式联运示范基地。

（三）围绕增活力，探索乡村振兴路径

乡村振兴开局良好。启动乡村振兴战略规划编制，在梁子湖区和华容区北部开展试点。大力实施“三乡”工程，能人回乡创办合作社、基地、企业528家。现代农业快速发展，农业增加值增长3%。划定粮食生产功能区和重要农产品生产保护区76万亩，新增“三品一标”农产品18个，“鄂州武昌鱼”“沼山胡柚”登陆央视。“虾稻生态繁育”获全国稻渔综合种养模式创新大赛一等奖。有效防范非洲猪瘟疫情。着力推进农旅融合，台湾农创园、石竹生态园、华兆山庄等田园综合体初具规模。

村居环境明显改善。加快推进美丽乡村建设，编制完成33个美丽乡村示范点规划，基本完成21个省、市美丽宜居示范村建设任务。扎实开展“四好农村路”提档升级、安全保障工程，大幅超额完成省下达计划。峒山村入选全国首批、全省唯一乡村环境综合治理示范村。实施“四个三”重大生态工程，新建、改建农户无害化厕所45713座、农村公厕259座；精准灭荒造林7602亩、防护林建设1200亩；建设集镇污水管网521公里，农村污水处理设施419个；探索推进生活垃圾分类试点。

农村改革卓有成效。稳步推进农村集体产权制度改革，全面启动清产核资、清人分类和股权量化工作。全面完成农村承包地确权登记颁证。完善承包地“三权分置”制度，创新股份合作、土地托管、委托经营等新的土地流转形式，流转耕地35.52万亩，流转率42.8%。在梁子湖区开展农村承包土地经营权抵押贷款试点，全年放贷1430万元，贷款余额3188万元。

（四）强力护生态，擦亮美丽鄂州底色

长江大保护成效显著。深入开展长江大保护十大标志性战役，重拳整治“散乱污”企业319家、畜禽养殖企业281家，拆除非法码头和水源保护区码头108个，腾退岸线10.8公里，建成砂石集运中心3个。加快恢复长江沿线生态环境，樊口区域沿江路及江滩环境综合整治、洋澜湖综合治理等项目顺利推进。梁子湖、花马湖实行全面禁捕。森林城市创建规划通过省级评审。

污染防治力度空前。综合治理扬尘、秸秆焚烧、机动车尾气等大气污染源，在重点行业执行特别排放限值，完成主城区油烟污染专项整治，全力打好蓝天保卫战。严格落实河湖长制，制定《鄂州市湖泊保护总体规划》《鄂州市湖泊保护实施方案》。洋澜湖43个污水排口全部封堵。开展农业面源污染防治，推进土壤污染详查，编制全市土壤污染治理与修复规划。

生态文明制度不断创新。建立环境治理和生态保护市场体系，推行生态文明绩效评价考核和责任追究制度，创新绿色考评体系，全面开展排污权、碳排放权有偿使用和交易工作。建立系统化生态价值体系，各区、开发区间横向转移支付生态补偿资金8286万元。这一探索创新入选改革开放40年地方改革创新40案例之一，相关经验被《人民日报》整版推介。连续两年在生态湖北创建工作考核中排名第三，梁子湖区成功创建全省生态文明示范区。

　（五）突出惠民生，提升群众幸福指数

精准脱贫扎实推进。围绕“两不愁、三保障”目标，强力推进脱贫攻坚。全市整合扶贫资金2.62亿元。全面实施健康扶贫“985”工程，扶贫对象实际人均住院报销比例达93.1%，居全省第一。农村贫困人口基本医保、大病保险、医疗救助、补充医疗保险“四位一体”工作机制进一步完善。白龙蓝莓入选全国首批、全省唯一的国家产业扶贫标准案例。产业扶贫为1.9万名贫困人员提供了增收渠道，金融扶贫、教育扶贫、兜底扶贫稳步推进，顺利通过中央脱贫攻坚专项巡视。

社会保障稳步提升。提高困难群众救助标准，城乡低保标准分别提高到580元/月、450元/月，特困供养人员标准提高到840元/月。3.8万低收入群体享受到价格临时补贴。大力发展“医养结合”养老模式，建设农村互助养老服务点207处、社区居家养老服务中心21处。加强社会保险扩面征收，深入推进机关事业单位养老保险制度改革。国有企业职工家属区“三供一业”分离移交工作居全省前列。

社会事业全面发展。新建、改扩建10所农村公办幼儿园，改造40所义务教育薄弱学校，完成鄂州职业大学汽车工程实训大楼、城东学校、恒大实验小学主体工程。获2017年度计划生育目标管理考评市（州）党政领导线一等奖，结核病防治经验全国推介，公立医院薪酬制度改革和健康校园建设工作经验全省推介。组织戏曲进校园，原创现代少儿京剧《童声》亮相第三届湖北艺术节，广场舞《凤凰飞旋》获省赛第一名。完成明堂体育场维修改造，成功举办2018鄂州国际半程马拉松赛。合理调整城市社区布局，开展社区物业管理提升年活动，完善老旧社区道路、消防通道等硬件设施。新增纯电动公交车60辆，规范交通文明行为，交通状况明显改观。

平安建设再创佳绩。深入开展扫黑除恶专项斗争，持续推进“霹雳行动”，摧毁黑恶势力团伙114个，推进“黄赌毒”“枪爆刀”“盗抢骗”“三堵五闹”等突出治安问题集中整治。全市社会治安明显好转，“一感两度两率”显著提升。车辆超载超限问题得到有效治理，亡人交通事故起数和死亡人数双下降。全市安全生产形势保持稳定，连续18年未发生重大食品药品安全事故。

我们不断加强政府系统党的建设和自身建设，有序推进机构改革各项工作。深化“放管服”改革，推行“马上办、网上办、就近办、一次办”和“最多跑一次”改革，统筹推进市级大数据中心和全市政务服务“一张网”建设，省考排名靠前。加强事中事后监管，推进全社会信用体系建设。加强法治政府建设，构建依法行政制度体系，制定《鄂州市物业管理办法》等11部规章，清理规章、规范性文件49件。加大《宪法》《监察法》等法律宣传普及力度。依法接受市人大及其常委会的法律监督和工作监督，社区物业管理工作接受市人大询问。主动接受市政协的民主监督。办理人大建议155件，政协提案238件。

外事、统计、审计、保密、工会、档案、气象、新闻出版广电、国防教育、民族宗教、防灾减灾、对外援助、妇女儿童、老龄、残联、人防、公共资源、社会科学等各方面工作都取得了新成绩。

各位代表，过去一年的成就，是我们认真学习贯彻习近平新时代中国特色社会主义思想和习近平总书记视察湖北重要讲话精神的结果，是市委坚强领导、科学决策的结果，更是全市干部群众齐心协力、共同奋斗的结果。在此，我代表市人民政府，向全市干部群众、各位人大代表、政协委员，向各民主党派、工商联、无党派人士、人民团体，向驻鄂州部队官兵，向所有参与、支持、关心鄂州建设发展的同志们、朋友们，表示衷心感谢和崇高敬意！

在成绩面前，我们要时刻保持清醒头脑，深刻认识到发展中还存在着一些问题和不足。经济发展方面，重大项目支撑不够，行业带动性强的大项目不多，在建项目推进不快；产业转型升级较慢，传统产业占比依然偏高；实体经济面临困难较多，特别是融资难、用地难、招工难问题依然存在；实体经济税收增长放缓，财政支出压力加大。社会民生方面，医疗、教育、社保、扶贫等工作还存在短板，与人民群众的期盼还有一定差距。生态环境方面，主城区大气治理任务依然艰巨，河湖港水污染整治与建设江南美丽田园城市目标差距较大。政府自身建设方面，少数干部素质、能力、作风与新形势新任务不适应，存在不想为、不会为、不敢为现象。我们一定正视发展中的困难，正视工作中的差距，不掩盖、不回避、不懈怠，以对人民高度负责的担当精神，在今后工作中切实加以解决。

　二、2019年工作安排

2019年是新中国成立70周年的大庆之年，是经济爬坡过坎的大考之年，是决战决胜全面建成小康社会的关键攻坚之年，也是我市深入推进城乡融合高质量发展的大干之年。面对我国经济运行稳中有变、变中有忧、外部环境复杂严峻的新形势，我们要牢牢把握习近平新时代中国特色社会主义思想这个根本遵循，牢牢把握以人民为中心这个根本目的，牢牢把握推动高质量发展这个根本要求，以稳中求进的姿态，应对稳中有变的环境，实现稳中向好的目标，全力以赴抓好今年工作。

今年政府工作的总体要求是：以习近平新时代中国特色社会主义思想为指导，全面贯彻党的十九大和中央经济工作会议精神，认真落实省委十一届四次全体会议暨全省经济工作会议精神，坚持稳中求进工作总基调，以供给侧结构性改革为主线，以建设现代化经济体系为目标，以改革开放创新为动力，以加快“三城一化”建设和乡村振兴为主要抓手，打好三大攻坚战，落实“六稳”工作部署，统筹推进“稳促调惠防”各项工作，在落实“四个着力”“四个切实”重要要求上取得新成绩，在贯彻省委“一芯驱动、两带支撑、三区协同”战略布局上取得新进展，保持经济持续健康发展和社会大局稳定，奋力谱写新时代鄂州城乡融合高质量发展新篇章，为全面建成小康社会收官打下决定性基础，以优异成绩庆祝中华人民共和国成立70周年。

辩证看待国际环境和国内条件变化，综合考虑各种因素，今年经济社会发展的主要预期目标是：全市地区生产总值增长8%左右，全社会固定资产投资增长11.5%，规模以上工业增加值增长8%以上，一般公共预算收入增长8.5%，社会消费品零售总额增长13%，高新技术产业增加值占GDP比重达到12.8%，居民收入增长与经济增长基本同步，全面完成省下达的节能减排任务，空气质量优良天数占比76%以上，主要地表水达到或好于Ⅲ类水体比例100%。

围绕以上目标，重点抓好八个方面的工作：

（一）坚持以三大攻坚战为突破口，着力夯实全面小康基础

防范化解财政金融风险。强化地方政府债务预算管理，规范推进政府购买服务、政府和社会资本合作，稳妥处理政府债务风险。深入开展互联网金融风险专项整治，严厉打击恶意逃废债务行为和非法集资、金融诈骗、非法“校园贷”等违法犯罪活动，加强对银行不良贷款的控制。深化投融资体制改革，调整优化国有资本投资运营模式，完成城投、昌达公司市场化转型。

打好精准脱贫攻坚战。按照“六个精准”要求，坚持政策标准，提高脱贫质量，进一步压实脱贫攻坚主体责任，做实驻村结对帮扶工作。大力推进产业就业扶贫“四个一批”工程，不断激发贫困群众脱贫致富的内生动力，引导社会力量参与脱贫攻坚，确保1.3万名贫困群众脱贫、5个省级贫困村出列，基本完成脱贫任务。

坚决整改环境保护突出问题。认真落实中央、省环保督察整改要求，不折不扣按期完成年度整改任务。加快葛店开发区生物医药企业搬迁及转型步伐，逐步解决工业区、居民区交织混杂问题。扎实做好城市黑臭水体治理、工业企业达标排放整治、畜禽养殖达标整治、“散乱污”企业整治“回头看”，推进“三高”企业和在用锅炉执行大气污染特别排放限值。启动球团厂搬迁计划，实施鄂钢节能环保工程3年计划，按超低排放标准，建设清洁、绿色、美丽的城市示范钢厂。

（二）坚持以项目建设为支撑，着力增强经济发展后劲

全面加快鄂州机场建设。推进机场工可和初步设计通过民航审批，全面完成机场开工前所有法定前置审批。启动机场主体工程建设，做好征地拆迁、矛盾调处等综合协调服务。加快配套项目建设，完成走马湖水系综合治理，启动机场安置小区二期工程，推进机场快速通道（S203鄂州段）、配套电力工程、污水处理等工程。完成机场航油码头及机场高速项目前期工作。

同步推进空港经济发展。启动机场核心区10平方公里空港经济产业园建设，加强与招商局集团等大型企业合作，围绕集成电路、高端装备制造、光电子信息、智能制造、新能源、新材料、人工智能、飞机改装和供应链管理等领域，导入一批大项目、好项目。成立空港产业基金、文化旅游产业基金等专项产业基金，强化重点项目资金支持。推进汀祖冷链产业园、航空物流产业园、枫树线业产业园、庙岭半导体产业园、葛店大健康产业园等园区建设。开展空港经济主题推介和国际路演，提高鄂州国际影响力。

着力强化项目投资工作。开展“项目建设年”活动，实行季度拉练、综合排名、首位奖励、末位约谈等制度，加大招商引资力度，加快项目落地建设。完善招商引资工作机制，优化考核奖励办法，形成全市招商引资一盘棋格局。力争引进省外资金300亿元，新签约亿元以上项目80个，新开工亿元以上项目65个。以项目配置资源，全力做好土地储备和出让，抓紧抓实项目规划、审批等前期工作，为重大项目建设提供要素保障。重点加快南都新能源二期、容百锂电四期、铠尔慷智能装备制造、虹润新材料扩建等一批重大工业项目建设。

（三）坚持以供给侧结构性改革为主线，着力构建现代经济体系

支持民营经济做强做优。落实好支持民营经济发展的各项政策措施，出台支持实体经济发展系列政策，进一步优化营商环境。构建亲清政商关系，保护企业家合法权益，打造公平竞争环境，给民营企业发展创造充足市场空间。充分发挥民营经济发展专项资金撬动作用，加强政府性融资担保体系建设，推动银行加大对实体经济的资金投放。加大企业上市服务力度，积极培育上市后备企业。新增省级隐形冠军10家、规模以上工业企业30家。

大力推进科技创新。深入推进“五链融合”，加快华科大鄂州工研院、中科院精密测量创新院等高层次创新平台建设，支持李时珍药物研究院建设，促进科研成果本地产业化。依托科技创新专项资金、股权投资引导基金和产业发展资金，加快湖北锐世、万度光能等项目的产业规模化进程。继续实施“新鄂州人”计划，加大科技人才的引进力度。支持企业产品创新，培育新产品30个以上，新增高新技术企业10家以上。实施百家企业技改工程，完成技改投资100亿元，重点支持传统产业装备改造、信息化智能化改造、节能安全环保改造。

加快现代服务业发展。推动先进制造业和现代服务业深度融合。重点支持仓储物流、临空服务、科技服务等现代生产性服务业发展，做大做强葛店千亿级电商基地，加快葛店国家级检验检测高技术服务业集聚区建设，推进华中万吨冷链港、潮团国际等项目建设。加强物流业管理体制机制创新，促进物流业提档升级。加快文化产业发展，培育新型文化业态和文化消费模式。推动红莲湖旅游综合体、融创体育小镇建设，打造一批旅游　　镇、名村和休闲示范农庄。启动西山4A级景区创建，探索梁子岛景区新的发展路径。积极推进服务业企业上规入库工作，力争服务业占GDP比重达到40%。

（四）坚持以全域规划为引领，着力构建城乡融合发展新格局

完善区域功能定位与空间布局。按照航空城、生态城、科学城和武鄂协同发展的路径，多规合一，编制鄂州市总体空间规划及各区、开发区片区规划、专项规划。全力推进机场核心区及毗邻片区开发，打造空港经济区；加快三江港现代物流新城、红莲湖文化旅游度假区建设；大力支持梁子湖区建设全国生态文明示范区，加快梧桐湖生态科学城建设步伐。做大做强葛店开发区光电子信息产业集群、新能源产业集群和电商基地，为全市经济发展扛大梁、作贡献。

实施城市精细化建设与管理。完成城市展示馆和美术馆工程建设。继续推进马鞍山、孙权大道地下综合管廊建设。完成鄂东大道和燕沙大道改扩建，启动鄂高西门立体畅通工程。加快新能源公交车替换，推进公交停保场和配套充电桩建设。继续实施“八点三线”交通整治工程，合理规划中心城区路外公共停车场布局，逐步解决城市道路交通堵点问题。完成城东水厂工程，实施城市照明工程。推进东洋澜湖湿地公园二期、华容公园、青天湖湿地公园、梁子湖环湖生态绿道等生态工程。加强城市精细化、信息化管理，建设智慧城市。做好国家卫生城市复查迎检工作，常态化推进全国文明城市创建。

推进武鄂协同和鄂咸黄黄联动发展。强化与武汉对接，统筹产业经济、生态保护、市政设施、公共交通、公共服务等综合布局，深入推进武鄂协同发展。推动新城大道、高新四路与武汉东湖高新区道路对接，加快鄂咸高速、武阳高速鄂州段、武汉地铁11号线葛店段建设。以空港经济为载体，促进鄂东转型发展示范区城市联动发展，建立常态化工作联系机制。

　　（五）坚持以实施乡村振兴战略为总抓手，着力推进农业农村现代化

加快推进试点示范。完成全市乡村振兴战略规划编制，加快梁子湖区、华容区北部试点示范。以农村居民市民化为方向，推进村湾集并和迁村腾地，盘活农村闲置宅基地和空闲土地，鼓励土地向集体经济流转，提升公共服务配置效率，加快城镇化进程，推进城乡融合发展。

培育壮大市场主体。加强政策引导，搭建小农户与现代农业对接平台，培育家庭农场、农民合作社等新型经营主体。积极探索多元化的集体经济发展路径，成立集体经济组织机构，充分发挥村集体自治组织和经济组织的双重属性，建立以村集体组织为依托的股份合作社或农业发展公司，发展壮大村集体经济。深入实施“三乡”工程，引导更多市场主体和能人下乡回乡，推动人员回归、资金回流、项目回迁。

切实振兴乡村产业。深化农业供给侧结构性改革，发展农业休闲观光体验、特色种养殖、农村电商等，延伸产业链条。培植壮大优势特色产业，形成城郊型、生态型多业融合格局。严格落实粮食安全行政首长负责制。持续做好非洲猪瘟疫情防控。强化优质农产品品牌创建，新认证“三品一标”产品10个。加快台湾农创园、峒山现代生态循环农业示范园建设，争创国家级台创园和田园综合体。

全面建设美丽乡村。学习推广浙江“千村示范，万村整治”成功经验，整合资金发挥效用，启动新一批“美丽乡村”试点建设，打造不同类型、不同发展模式的示范村，培育自然生态、人文历史、民居风貌、民俗文化等特色村。加强村湾环境整治，大力推进“四好农村路”、民居改造、绿化亮化等农村人居环境工程。

　（六）坚持以绿色发展为遵循，着力建设美丽鄂州

深入开展长江大保护和湖泊生态修复。强力推进长江大保护十大标志性战役和绿色发展十大战略性举措，在长江大保护中展示鄂州担当作为。严格环境准入，加强规划环评和建设项目环评管理。严格落实河湖长制和《鄂州市湖泊保护条例》，促进全市湖泊、河流、水库水质全面改善。加快梁子湖湖泊治理国家示范区创建，推进江湖连通、湖湖连通和樊口区域沿江路及江滩环境综合整治、洋澜湖综合治理等工作，建成樊口二站。启动长港河、高桥河、新港治理，完成鸭畈港、新农村港治理。做好矿山地质环境修复和综合治理。

深入推进“四个三”重大生态工程。加快五类厕所建设，提前一年完成“厕所革命”任务。积极创建省级森林城市，义务植树200万株，精准灭荒造林3800亩，长江岸线造林4700亩。扎实做好松材线虫病防控工作。全面完成污水全收集全处理全覆盖，完成城区污水处理厂扩容，启动葛华污水处理厂三期和樊口污水处理厂提升工程建设。推广生活垃圾分类，完善生活垃圾收运体系，力争到2020年城市和农村生活垃圾无害化处理率分别达99％、90％以上。

深入推进生态文明体制改革。稳妥推进环保监测监察执法垂直管理改革，健全机构、加强力量、理顺体制。探索推进生态价值实现机制试点工作，实施生态价值指标交易制度，建立生态价值测算评估体系，加大生态融资创新力度，大力发展绿色金融。实现自然资源资产负债表编制和领导干部自然资源资产离任审计常态化，积极推进排污权交易和有偿使用。

（七）坚持以改革开放为动力，着力再造高质量发展新机制

深化政府机构改革。优化政府机构设置和职能配置，构建系统完备、职责明确、科学规范、运行高效、依法行政的现代化政府治理体系。推进区、开发区、街办管理体制改革，理顺市、区财政事权和支出责任。加快构建城乡融合高质量发展的规划体系、指标体系、考核体系、保障体系。加强预算管理，完善预算绩效管理体系。

深化“放管服”改革。接力推行“马上办、网上办、就近办、一次办”，加强政务服务“一张网”、基层便民服务中心和大数据中心建设，简化公共服务流程。强力推进投资项目审批改革，减少环节、优化流程、提高效率，加强事中事后监管，激发市场活力和发展内生动力。

提升对外开放水平。加快空港综合保税区前期工作，编制综合保税区产业规划和机场口岸规划。力争鄂州空港口岸纳入国家口岸开放规划。积极对接武汉自贸区，依托鄂州机场、三江港，加快打造“公铁水空”多式联运体系，提升空港枢纽功能。加强外向型经济培育，积极融入“一带一路”建设。

（八）坚持以满足人民对美好生活的向往为目标，着力保障和改善民生

做好就业和社会保障工作。多渠道扩大创业就业，加大培训力度，扶持城乡困难人员、残疾人就业，动态消除零就业家庭，城镇新增就业1.9万人以上，城镇登记失业率控制在4%以内。切实做好退役军人服务工作。完善社会保障体系，提升社会救助水平，保障特困群众基本生活。大力推进“医养融合”，提高养老机构水平和质量。继续推进城南、英山、月陂、茨塘、菜园头等安置小区建设。

大力发展社会事业。优先发展教育事业，新建城南学校、花湖学校，加快西山小学、市二中、葛店高中迁建，解决城区义务教育大班额问题；新建、改扩建一批标准化幼儿园，解决学前教育入园难问题；推进长江职院新校区建设，促进社会培训机构健康发展，提高职业教育水平。合理布局生态公墓，改造提升团山公墓，加强殡葬市场治理。推动公共文化体育服务基础设施提档升级，改扩建市群艺馆新馆、市非遗馆，维修改造鄂州大剧院，加快区级“三馆一场”建设，加强公共文化体育设施维护管理。推动全民阅读，建设“书香鄂州”，提升市民科学素质。扎实推进健康鄂州建设，启动儿童医院建设，实施全科医生委培、中医药人才培养“双百”计划。推进基层医疗卫生机构改革，推进乡镇卫生院提档升级，打造城区15分钟服务圈。

加强和创新社会治理。继续实施社区建设“40工程”，创新城市社区服务管理模式，持续推进物业管理提升三年行动计划。加强农村基层社会治理。全面深化公安改革，深入推进扫黑除恶专项斗争，保持“霹雳行动”常态化，深化平安鄂州创建。推进立体化信息化治安防控体系“186”工程建设，加快派出所标准化、“两中心一体化”、智慧平安小区等重点项目建设。深入推进公共法律服务体系建设。做好第四次全国经济普查。强化市场监管，维护消费者权益。建立应急管理机制，推进城市安全风险防控，加强安全生产隐患排查，深入推进打非治违专项行动，坚决遏制较大安全事故。实施食品安全战略，让群众吃得放心。

　三、加强政府自身建设

新时代要有新气象、新担当、新作为。我们将始终不忘初心、牢记使命，不断提高政府治理现代化水平，努力建设人民满意的服务型、法治型政府。

　（一）旗帜鲜明讲政治。牢固树立“四个意识”，坚定“四个自信”，严守政治纪律和政治规矩，坚决维护习近平总书记核心地位，坚决维护党中央权威和集中统一领导。严格党内政治生活，推进“两学一做”学习教育常态化制度化，扎实开展“不忘初心、牢记使命”主题教育。坚决贯彻落实党中央国务院、省委省政府和市委的各项决策部署，确保政令畅通。

（二）依法行政促公正。强化法治思维，加强重点领域政府规章制定，加强层级监督，严格规范公正文明执法。自觉接受人大法律监督和工作监督、政协民主监督、监委国家监察，主动接受社会监督、舆论监督，高质量办好人大建议和政协提案，接受人大询问，推进决策科学化、民主化、法治化。全面推进政务公开，加强舆情应对，主动回应社会关切。

　（三）真抓实干转作风。开展作风建设三年行动，坚决治理形式主义、官僚主义顽症。大兴调查研究之风，坚持一线工作法，察实情、体民忧、解难题。完善政府工作推进、协调、督查体系，转文风、改会风，强落实、重实效，以钉钉子精神确保每一项工作、每一条措施都落到实处。切实减轻基层负担，让基层把更多时间用在抓落实上。进一步健全制度，激励干部增强干事创业的精气神，让真抓实干成为政府工作的主旋律。

（四）廉洁从政葆本色。切实履行全面从严治党主体责任和“一岗双责”，大力开展反腐倡廉，严格落实中央“八项规定”及实施细则精神和省委、市委相关规定，坚定不移纠“四风”、树新风。深化廉政风险防控，不断健全“不敢腐、不能腐、不想腐”的体制机制，做到敢管敢严、真管真严、长管长严。加强纪律教育，强化纪律执行，努力营造政府系统风清气正的政治生态。

各位代表！潮起海天阔，扬帆正当时。鄂州正处于改革发展的关键时期，做好今年的工作意义重大，任务艰巨。让我们紧密团结在以习近平同志为核心的党中央周围，在省委、省政府和市委的坚强领导下，紧紧依靠全市人民，真抓实干、埋头苦干、少说多干，为鄂州城乡融合高质量发展而努力奋斗！
